# Hydrus Microstent malposition with uveitis-glaucoma-hyphema syndrome^☆^

**DOI:** 10.1016/j.ajoc.2022.101405

**Published:** 2022-02-09

**Authors:** Cara E. Capitena Young, Deidre M. St Peter, Monica K. Ertel, Mina B. Pantcheva

**Affiliations:** Department of Ophthalmology, Sue Anschutz-Rodgers Eye Center, University of Colorado School of Medicine, Aurora, CO, USA

**Keywords:** Hydrus, Implant malposition, Uveitis-glaucoma-hyphema syndrome

## Abstract

Intraocular implants, specifically those used in the treatment of glaucoma, are each associated with various implant related risks and complications of which surgeons placing these devices must be aware. Here we present a case of uveitis-glaucoma-hyphema (UGH) syndrome associated with the Hydrus Microstent.

## 1 Case report

**Fig. 1:**
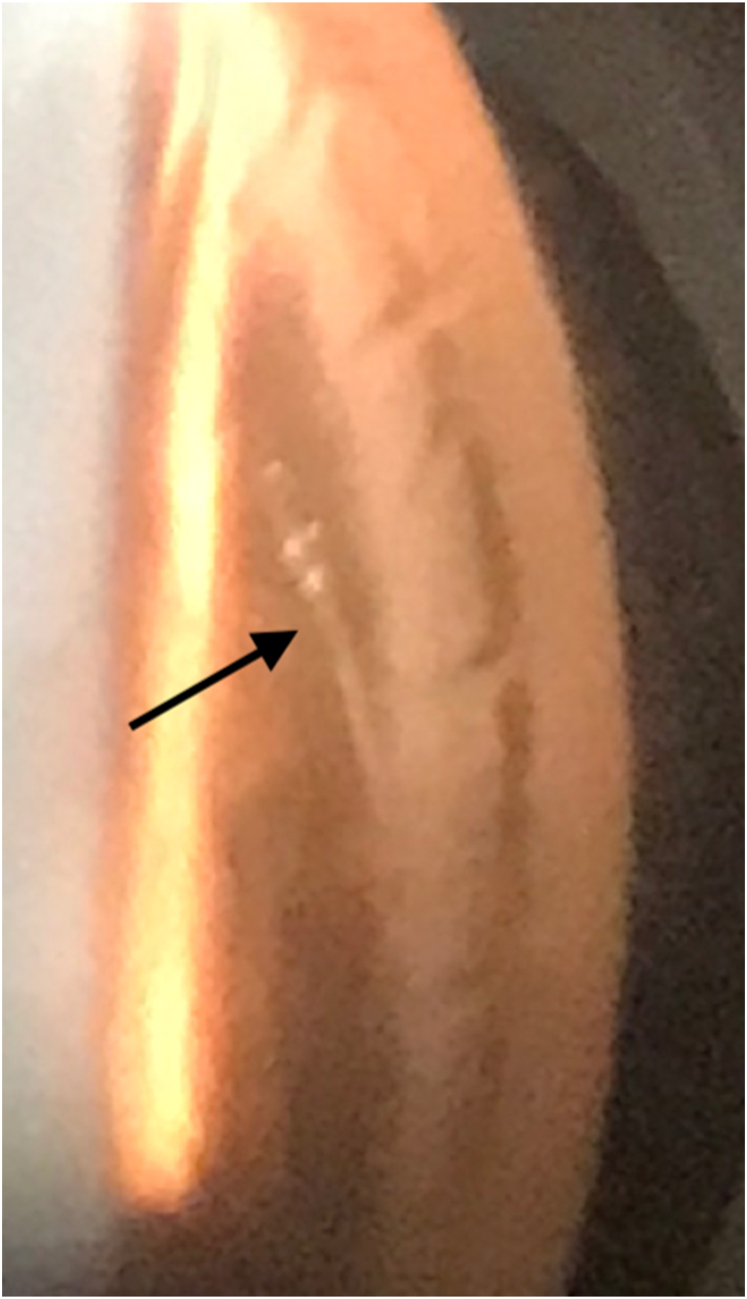
Portable slit lamp photo of the nasal angle under gonioscopic view (through corneal edema and heavy mixed anterior chamber cell) showing proximal end of the Hydrus implant (black arrow) outside of the canal of Schlemm, lying on the iris.

A 61-year-old male with a history of primary open angle glaucoma was referred for persistent, severe eye pain starting immediately after uncomplicated phacoemulsification with intraocular lens implantation and Hydrus microstent placement in the left eye 4 weeks prior. His pain did not improve with the standard post-operative steroid and nonsteroidal drop regimen. He was referred to an oculoplastic surgeon who recommended periocular steroid injection and an oral steroid course for presumed supraorbital neuralgia. This improved the pain for 2 days after which his symptoms recurred along with worsening photophobia. He returned to his primary surgeon where exam of the operative eye showed acuity of 20/200 and an intraocular pressure (IOP) of 40 mmHg. Slit lamp exam described 3+ corneal edema and a deep and quiet anterior chamber (AC). His IOP was treated with maximum topical IOP-lowering drop therapy and oral acetazolamide and he was referred to University of Colorado for additional opinion. At this time, his left eye visual acuity was 20/70, IOP was 24 mmHg with 2+ afferent pupillary defect. Slit lamp exam revealed 3+ mixed AC cell and nasal transillumination defects. The Hydrus implant was partially visible at the slit lamp nasally. On gonioscopic examination, the snorkel and first window of the implant were noted to be outside of Schlemm's canal, lying on the nasal iris adjacent to the transillumination defects (Fig. 1). The distal portion of the implant was not visible under anterior synechiae. Given the implant malposition and suspicion for uveitis-glaucoma-hyphema (UGH) syndrome as the etiology of his pain and IOP spike, the implant was removed with almost immediate and persistent resolution of pain and gradual improvement of the AC reaction. After Hydrus removal, the patient required further filtering surgery for pressure control.

## 2 Discussion

The Hydrus Microstent (Ivantis, Irvine, CA) has shown promising results in regards to its IOP lowering abilities,^1^ thus making it an appealing treatment for patients with open angle glaucoma. Currently, the Hydrus is one of several intraocular implants available on the market. Initial safety results from the HORIZON trial reported 5 cases (1.4%) of device malposition of which none required surgical removal or repositioning. Other pertinent adverse events included 2 cases (0.5%) of persistent uveitis, 40 cases (10.8%) of anterior synechiae and 27 cases (7.3%) of partial or complete device obstruction.^1^ There were also 6 cases of inflammation, 4 instances of iris-stent touch, a 1 case of hyphema associated with the Hydrus Microstent reported to the Food and Drug Administration Manufacturer and user Facility Device Experience (MAUDE) database between January 2009 and December 2019.^2^ There are similar cases of device malposition and associated complications with other intraocular devices such as the iStent (Glaukos, San Clemente, CA) and the ExPress (Alcon, Fort Worth Texas) as well.^3^^,^^4^ Because of the potential secondary ocular effects it is prudent to be aware of the potential complications of any intraocular implant.

## 3 Conclusion

All ocular implants carry with them unique risks of which all surgeons must be aware. Complications include but are not limited to implant migration, secondary synechiae, persistent uveitis, UGH syndrome, corneal endothelial damage and device obstruction.^2^ In any patient with an implant who has persistent inflammation, bleeding, iris transillumination defects, or pain; implant malposition and the associated secondary sequelae should be considered and the position of the implant should be evaluated carefully.

## Funding

No funding was received for this report.

## 4 Authorship

All authors listed contributed to the care of this patient. All authors attest they meet the current ICMJE criteria for authorship.

## Declaration of competing interest

The authors have no relevant conflicts of interest to disclose:CECY, DMS, MKE, MBP.
